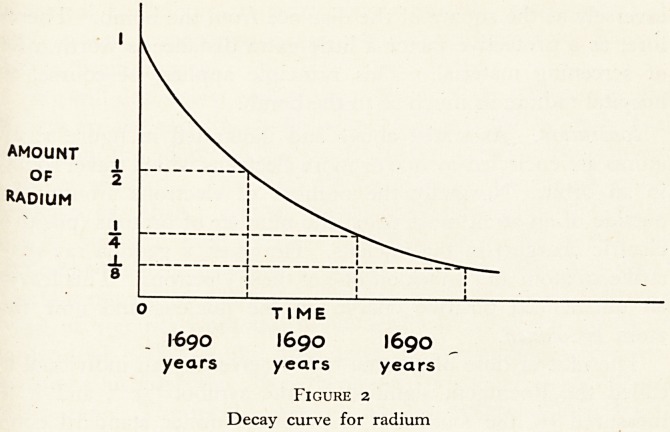# The Release of Atomic Energy and the Pathological Effects of Radiation

**Published:** 1951-07

**Authors:** R. C. Tudway

**Affiliations:** Radiotherapist, United Bristol Hospitals


### The Bristol Medico-Chirurgical Journal

*A Journal of the Medical Sciences for the*

### West of England and South Wales

*" Scire est nescire, nisi id me*
*Scire alius sciret."*

JULY, 1951

### THE ATOMIC BOMB*

### I: THE RELEASE OF ATOMIC ENERGY AND THE PATHOLOGICAL EFFECTS OF RADIATION

BY

R. C. TUDWAY, M.B., B.S., B.Sc., D.M.R.
*Radiotherapist, United Bristol Hospitals*

An outline of the scientific background behind the explosive release of atomic energy should be not only of general interest but also of practical assistance. It should help doctors to anticipate the effects of the atomic bomb in circumstances not exactly covered by the practical account, or to follow accounts of new ideas yet to be published.

The atoms of the various chemical elements are each composed of an atomic nucleus in the centre, and one or more electrons circulating round the nucleus like the moon round the earth. The nucleus is very small and relatively heavy. The electrons are very light and each one carries one negative charge of electricity. The nuclei of atoms are always built up of two kinds of particles, protons and neutrons, in different proportions.

* Papers read at a meeting of the Bristol Medico-Chirurgical Society on Wednesday, 10th January, 1951.

Protons are atomic particles which carry one positive charge of electricity and have a mass of one unit. Neutrons have a mass of one unit but no electric charge.

Figure 1 illustrates this by showing three kinds of hydrogen atoms. The variety of an atom is determined by the number of protons (*i.e.* positive charges) in its nucleus. So all these atoms are hydrogen, because they all have only one proton in the nucleus, one proton being characteristic of hydrogen. But these three atoms are different, because they have different numbers of neutrons: and they are called different isotopes of hydrogen. The total mass of the nucleus in each case consists of the number of protons and neutrons added together and is called the mass-number of the atom.

(*a*) The ordinary hydrogen atom $H^{1}_{1}$: 1 $+^{ve}$ charge of electricity and 1 unit of mass in the nucleus.

(*b*) Deuterium or Heavy Hydrogen $H^{2}_{1}$: 1 $+^{ve}$ charge and 2 units of mass in the nucleus.

(*c*) Tritium $H^{3}_{1}$ (radioactive): 1 $+^{ve}$ charge and 3 units of mass in the nucleus.

FIGURE 1

*Radioactivity.* As well as illustrating the occurrence of isotopes, figure 1 illustrates the occurrence of radioactivity. The nuclei of atoms are not composed of protons and neutrons gathered together in any proportions: only a few of the many possible combinations of protons and neutrons will in fact stick together. In the lighter elements about equal numbers of each are needed to form a stable nucleus, and in the heavier atoms about two protons for every three neutrons. An atom of *Tritium* or $H^3$, which has two neutrons to only one proton, tends to fly apart, *i.e.* it is radioactive (Figure 1).

The heaviest atom which is *stable* is that of Bismuth with 209 protons and neutrons in the nucleus, *i.e.* $B^{209}$. All atoms heavier than this are unstable or in other words radioactive: for example, $Ur^{235}$, $Ur^{238}$ and $Ur^{239}$, the three natural isotopes of uranium, which is the heaviest natural element. During ordinary radioactivity the nucleus of an unstable atom does not split into two halves. It merely keeps on losing small particles until it reaches a stable condition. The types of particles it emits are:

1. *Alpha particles.* These have a mass of four, and two positive charges on each.

2. *Beta particles*, with a very small mass and one negative charge. These are accompanied by

3. *Gamma rays*, which are most important and responsible for most of the radiation casualties from the atomic bomb.

### Atomic Fission

There is such a thing, however, as a splitting of the atoms. This is Atomic Fission, discovered in Berlin in 1939 by Hahn and Strassmann. There are everywhere a few stray neutrons moving about. If a $Ur^{235}$ atom is struck by one stray neutron it absorbs this neutron and becomes for an instant $Ur^{236}$. The $Ur^{236}$ atom cannot exist for more than an instant however. It splits down the middle and this produces: (*a*) two smaller atoms, *e.g.* Barium and Krypton (or many other pairs); (*b*) three or four spare neutrons, and (*c*) very much energy. If a mass of $Ur^{235}$ atoms is present this effect is propagated, due to the spare neutrons setting off more Ur atoms and so on. This is the "chain reaction": within a fraction of a second it has built up into:

*The Atomic Explosion.* Most of the fission products are not natural elements and so are extremely radioactive. They produce rays which are very intense for about ten seconds. But unless the $Ur^{235}$ is pure this explosion will not happen because the impurities waste all the neutrons. To make an atom bomb, therefore, all that is needed is to collect a large enough mass of pure $Ur^{235}$—a very difficult thing—and to bring the mass together only when an explosion is needed, since a mass smaller than a certain critical size will not explode. $Ur^{235}$ occurs as only 0·7 per cent. of natural uranium, most of which is $Ur^{238}$.

In practice, another element, plutonium made from $Ur^{238}$ in the atomic pile is now used. The great energy of the bomb is due to the fact that during atomic fission some of the mass of the uranium is converted into energy.

This is shown by the formula: $E = MC^2$

Where $E$ = energy formed; $M$ = the mass lost ; $C$ = the velocity of light $= 3 \times 10^{10}$ cms./sec., so that $C^2 = 9 \times 10^{20}$, a very big number: and the energy E is enormous.

### The Decay of Radioactive Material

As the atoms of any radioactive material are constantly breaking down, it must be steadily disappearing. This is illustrated by figure 2 which is the decay curve for radium. It shows that in 1,690 years the radium one starts with has decayed to half. At the end of another 1,690 years, one-half of this remaining half has gone, *i.e.* one-quarter remains. At the end of a third 1,690 years only one-eighth and so on, but the radium never entirely disappears. This is expressed by saying that its " half-life " is 1,690 years (Figure 2).

Fortunately the half-life of the fission products of the bomb is mostly very much shorter, and is measured in seconds rather than years. But some long-lived elements are formed which fade away very slowly until negligible amounts are left.

We must now consider the properties of the gamma rays emitted by the fission products during and after the atomic explosion. Alpha, beta and neutron rays will be ignored now as they do not in practice produce many casualties. The properties of gamma rays which matter to us are:

1. Penetration.
2. "Half-value-layer."
3. Straight line path—inverse square law.
4. Ionization.

FIGURE 2
Decay curve for radium

*Penetration.* The gamma rays from the bomb have very great powers of penetration. They traverse 3½ inches of concrete before they are reduced to half-strength. Alternatively 0·6 inches of lead, 1 inch of steel or 8 inches of water all reduce them to half-strength. This means that people within buildings are only very incompletely protected from gamma rays.

" *Half-Value-Layer*". The strength of gamma rays falls after penetrating an increasing thickness of concrete. The first 3½ inches traversed reduces the gamma rays to half the original strength, the next 3½ inches reduces this half to half again, *i.e.* to one-quarter the original, the next 3½ inches reduces the rays to one-eighth and so on: but no thickness will cut them out entirely. The thickness of any substance needed to cut the gamma rays to half is called the " half-value-layer " of the substance: *e.g.* 3½ inches of concrete or 1 inch of steel. All this means that considerable thicknesses are needed to shield effectively against the rays.

*Straight Line Path.* Gamma rays are similar to light rays: they only travel in straight lines. This has one important effect. The beam of rays to which an individual is subjected will broaden out like the beam from a headlamp. So the area covered by the beam of rays increases with the square of the distance from the source, and the intensity of radiation varies inversely as the square of the distance from the bomb. Therefore, as a protective factor a little extra distance is worth a lot of screening material. This principle applies, of course, to hospital radium as much as to the bomb.

*Ionization.* As stated above and illustrated in figure 1, all atoms are encircled by one or more electrons which travel round in an orbit. Normally the number of electrons around the outside of an atom must equal the number of protons (positive electric charges) in the nucleus. However, a gamma ray may strike an atom and knock off one of these electrons. This leaves an unbalanced positive charge on the nucleus and now the atom is *ionized.*

The unit of dose of gamma rays received by an individual is called the Roentgen, signified by the symbol " r ", and it is measured by the ionization it produces under standard conditions. The effect of so many r falling on an individual depends not only on the number of r on each square centimetre, but also on the size of the part irradiated. Exposure to radiation from the atom bomb, however, nearly always involves the whole body: the effects of dosage will be given later on that assumption.

Ionization is also responsible for the biological effects of radiation. The molecules of body substances are changed by it. The effects appear to be purely destructive, and there is no evidence of a stimulating effect. The effects of radiation are, however, selective, for the sensitivity of different tissues to ionization varies. For example, in the treatment of radiosensitive growths the normal cells are less easily killed than the malignant. And in the case of irradiation of whole animals by the atomic bomb or by experiment the effects are still selective: some tissues are more sensitive than others, also different animals differ in their sensitivity.

For example, for gamma rays:

200r kills 50 per cent. of guinea pigs.

500r kills 50 per cent. of men.*

800r kills 50 per cent. of rabbits.

The tissues themselves are affected in the following order of sensitivity which is similar for all animals: lymphatic tissue,

* This is the maximum of a suggested range of 300–500r based on observations in Japan.

germinal epithelium of testis, bone marrow, gastrointestinal, ovary, skin, connective tissue, bone, liver, pancreas, kidney, nerve, brain, muscle.

We will deal below with the first few of these. Effects on the others are not seen in whole-body-irradiation, as the individual dies from the effects on the sensitive tissues before the resistant can be affected. The fatal effects of radiation are threshold effects: less than a certain dose per day or week will have no effect however long continued. The threshold varies for different tissues. Recovery from radiation damage does occur but it is not always complete. It is thought that up to a tolerance dose of 0·5r per week can be accepted without damage by an individual. This is the dose used for calculating safety precautions in hospitals. Non-threshold effects do occur, but are more rare. The most important are the genetic effects due to direct hits by the radiation on the genes. I propose to say nothing about these. Little is known of their importance, and even in the populations of Hiroshima and Nagasaki several generations may be needed for the effects of mutations to appear. As well as direct action by ionization within the cell producing damage there is some evidence for humoral effects involving the production of diffusable toxic substances, both in local effects and the general radiation sickness.

### The General Effects of a Single Large Exposure to Gamma Rays

We will now concentrate on the acute radiation effects of the single large dose which comes from the explosion of the bomb. The effects of chronic dosage are concerned with more peaceful hazards, except if there should be an area of ground contamination from the bomb and essential work has to be carried on by people within the area.

There are three groups of fatal cases:

1. Shock may cause death in five to forty-eight hours.

2. Toxaemia may cause death in five to fifteen days (ten to fifteen days mostly), after initial improvement, especially following the mid-lethal dose of 400–500r.

3. Haemopoeitic damage may cause death in thirty to ninety days.

The toxaemic effects are:

(*a*) *Fever.* A step-ladder temperature sometimes going on to hyperpyrexia and death, commencing two to twenty-eight days after exposure. In non-fatal cases this usually starts in the third week, at about the same time as epilation.

(*b*) A Heparin-like substance is produced, causing loss of clotting power and increased bleeding time, an important factor in severe cases dying within seven days.

The more commonly seen haemorrhagic state occurs at three to four weeks and is associated with capillary damage. After the first seven days the commonest cause of death is agranulocytosis, aggravated by the fever and haemorrhages. The effects on the blood forming organs have been most studied and are of most interest. The lymphocytes are the most sensitive of all cells. They reach a minimum, in the blood, in about three to five days and may nearly completely disappear after a heavy exposure. The germinal centres of the lymph nodes disappear, but recovery begins very soon afterwards. The blood-forming elements of the bone-marrow are damaged and in severe cases nearly disappear (except in small islets) within two weeks. Owing to hyperaemia the marrow remains red. Recovery usually begins before four weeks in a non-fatal case. The circulating granulocytes reach a minimum at about six to fourteen days after exposure. The platelets reach their minimum about one week later and the red cells after about twenty-one days. The anaemia may be very slow to recover, taking several months. The sedimentation rate rises after one week.

*The Gonads.* Germinal epithelium and developing ova are very sensitive and temporary sterility is common. They are, however, replaced after a varying interval in nearly all surviving cases, and the dose of total body radiation needed to cause permanent sterility is more than the fatal dose.

*Gastro-Intestinal Epithelium.* Oedema sometimes followed by false membrane formation occurs after two to three days in severe cases. Ulceration and submucous haemorrhages are also produced.

*The Skin.* Erythema is not likely to be seen as it needs a dose well above the lethal dose (up to 1,000r).

*Epilation* is often seen, and it has been found in Japan that cases which are not epilated usually recover. It occurs towards the end of the third week.

Effects due to the ingestion of radioactive elements may be met with and if they do occur can be most serious, especially when elements, *e.g.* radioactive strontium or plutonium, are deposited in the bones. This may result in bone-marrow damage long afterwards, *i.e.* anaemia and agranulocytosis, or sometimes in leukaemia or osteogenic sarcoma.

## Figures and Tables

**Figure 1 f1:**
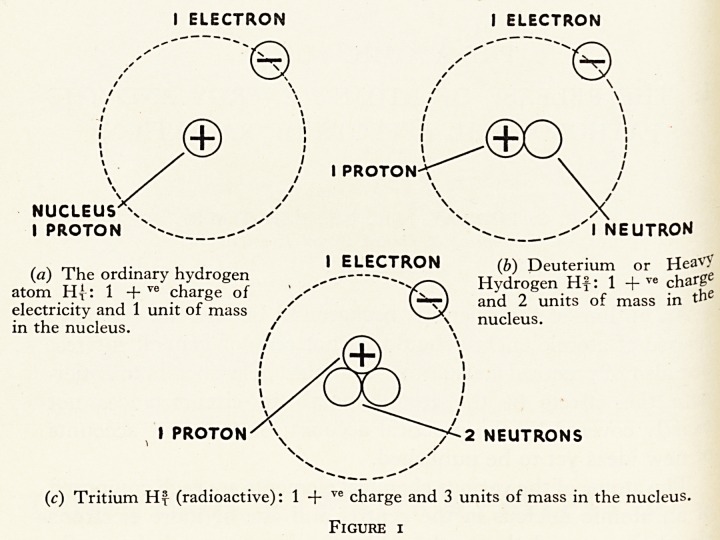


**Figure 2 f2:**